# Prevalence and stability of mental disorders among young adults: findings from a longitudinal study

**DOI:** 10.1186/s12888-018-1647-5

**Published:** 2018-03-12

**Authors:** Kristin Gustavson, Ann Kristin Knudsen, Ragnar Nesvåg, Gun Peggy Knudsen, Stein Emil Vollset, Ted Reichborn-Kjennerud

**Affiliations:** 1 Department of mental disorders, Norwegian Institute of Public Health, Oslo, Norway; 2 Centre for Disease Burden, Norwegian Institute of Public Health, Bergen, Norway; 3 Department of Psychosocial Science, University of Bergen, Bergen, Norway; 4 Nydalen DPS, Division of Mental Health and Addiction, Oslo University Hospital, Oslo, Norway; 5 Department of Psychology, University of Oslo, Oslo, Norway; 6 Health Data and Digitalisation, Norwegian Institute of Public Health, Oslo, Norway; 7 Institute of Clinical Medicine, University of Oslo, Oslo, Norway

**Keywords:** Mental disorders, Young adulthood, Health surveys, Prevalence, Stability

## Abstract

**Background:**

Mental disorders often have onset early in life, contribute substantially to the global disease burden, and may interfere with young people’s ability to complete age-relevant tasks in important developmental periods. However, knowledge about prevalence and course of mental disorders in young adulthood is sparse. The aim of the current study was to estimate prevalence and stability of mental disorders from the twenties to the thirties/forties.

**Methods:**

DSM-IV mental disorders were assessed with the Composite International Diagnostic Interview in two waves (1999–2004 and 2010–2011) in 1623 young adult Norwegian twins (63.2% women, aged 19–29 years in wave 1).

**Results:**

In wave 1, the 12-month prevalence of any mental disorder among people in the twenties was 19.8% (men) and 32.4% (women), anxiety disorders: 9.6% (men) and 26.7% (women), anxiety disorders excluding specific phobias: 2.5% (men) and 6.9% (women), major depressive disorder (MDD): 4.4% (men) and 7.2% (women), and alcohol use disorder (AUD): 8.7% (men) and 4.4% (women). The prevalence of any mental disorder decreased from the twenties to the thirties/forties. This was due to a decrease in AUD and specific phobias. Anxiety disorders in the twenties predicted anxiety disorders and MDD ten years later, even when controlling for the association between these disorders in the twenties. MDD in the twenties predicted MDD ten years later. At both ages, two-week and 12-month prevalence estimates differed markedly for MDD - indicating an episodic course.

**Conclusions:**

Common mental disorders are highly prevalent among young adults in the twenties, and somewhat less prevalent in the thirties/forties. Those who suffer from one mental disorder in the twenties are at considerably increased risk for suffering from a disorder ten years later as well. This may have significant implications for young people’s ability to attain education, establish a family, and participate in occupational life.

**Electronic supplementary material:**

The online version of this article (10.1186/s12888-018-1647-5) contains supplementary material, which is available to authorized users.

## Background

Mental disorders are among the most prevalent health problems affecting the adult population [1]. The Global Burden of Disease Study 2015 (GBD 2015) estimated that seven of the top 25 causes of years lived with disability (YLD) globally were mental disorders, with major depressive disorder (MDD) ranked second and anxiety disorders ranked ninth [2]. The high rankings were a result of both high prevalence and the disability associated with these disorders. Estimates of 12-month prevalence of mental disorders vary between 9.6 and 27.8% in the general adult population [3–8]. Mood disorders, anxiety disorders (including specific phobias), and alcohol use disorders (AUD) are the most prevalent disorders and are included in all the above-mentioned studies.

### Young adulthood

The shift in recent decades toward longer educations and higher age when committing to stable relationships and having children, has led researchers to aknowledge the age between 18 and 29 as an important developmental period [9]. It is characterized by changes in love and work life, and frequent residental change [9, 10]. Completing education and building a stable life structure are important developmental tasks in this period, while raising children is an important task for later periods [9, 10].

Mental health problems are associated with poor educational attainment as well as work and interpersonal problems [11–18]. Research results indicate that half of US adolescents’ failure to complete secondary school is attributable to mental disorders [19]. Mental disorders are also associated with maladaptive parenting behaviors, such as low affection toward the child and inconsistent enforcement of rules [20]. Because mental disorders may interfere with developmental tasks in different periods, we need to increase our knowledge about the prevalence and stability of such disorders at different ages to be able to correctly assess the magnitude of this problem.

### Prevalence of mental disorders in young adulthood

Findings from the New Zealand Mental Health Survey (NZMHS), the Dutch Tracking Adolescents’ Individual Lives Survey (TRAILS), the US National Comorbidity Survey (NCS) and its replication (NCS-R), and the Children in the Community Study (CICS) from the US, show 12-month prevalence estiatmes of MDD between 8.3 and 12.4% among people between 18 and 33 years [16, 21–23]. The 12-month prevalence was between 19.4 and 22.3% for anxiety disorders (including specific phobias), between 2.5 and 10.3% for acohol dependence, and between 7.1 and 18.4% for alcohol abuse [21–23].

### Stability of mental disorders in young adulthood

Previous studies indicate that the prevalence of depression is stable over the earliest part of adulthood, whereas AUD decreases, and findings regarding anxiety disorders are mixed [21, 24]. In the Dunedin study from New Zealand, the 12-month prevalence of MDD was 16.8% at age 21 years and 16.3% at 32 years [21]. The 12-month prevalence of any anxiety disorder (including specific phobias) was 20.3% at 21 years and 22.2% at 32 years. In the Simmons Longitudinal Study from the US, prevalence estiamtes of MDD showed a weak and not statistically significant increase from age 21 to 30 (from 5.4 to 8.0%), while there was a decline in the 12-month prevalence for phobia (including specific phobias) (from 16.8 to 2.3%) [24]. The prevalence of alcohol dependence declined (from 18.4 to 8.1%) from age 21 to 32 in the Dunedin study, and the prevalence of AUD declined from 26.7 to 6.0% in the Simmons Longituidnal Study [21, 24].

Some people have long-lasting and/or recurrent episodes of mental disorders [25, 26]. In the general adult population, about 40% of those who have depression or anxiety at one time point, have been found to have at least one of these disorders 7 years later [27]. Having a disorder or mental health problems in adolescence increases the risk of such disorders/problems in early adulthood [18, 28–30]. Findings from the NCS follow-up (NCS-2) showed that having MDD or generalized anxiety disorder (GAD) in the twenties and thirties predicted having any of these disorders eleven years later [31]. Depression seems to be more episodic than anxiety disorders among adolescents, as shown by higher 12-month to 30-day prevalence ratios of mood than anxiety disorders [22].

### Comorbidity

Comorbidity between mental disorders is extensive with up to 50% of those who have one mental disorder also having at least one additional comorbid mental disorder [4, 7, 32]. This is also reported among young people [33]. Comorbidity seems to a large degree to be due to common liablity factors for several disorders [34, 35] and is related to severity and chronicity [27, 33, 36, 37]. Hence, estimates of comorbidity in different developmental periods may increase our understanding of the potential effects of common mental disorders on young adults.

### Need for further knowledge

Current knowledge on prevalence and stability of mental disorders among young adults relies heavily on results from the US and New Zealand. Longitudinal studies from several different countries are needed to inform policy makers in different parts of the world and to estimate global disease burden in this age period.

Previous prevalence estiamtes of any mental disorder or any anxiety disorder typically include specific phobias, which by definiton involves imapirment in circumscribed situations and can sometimes be treated in a single therapy session [38]. Prevalence estiamtes both including and excluding such phobias are therefore needed to plan mental health services for young adults.

Findings are divergent with regards to whether or not anxiety disorders become less prevalent during young adulthood. Previous studies have included different anxiety disorders when examining this. There is a lack of studies tracking the episodic versus chronic nature of MDD and anxiety disorders across young adulthood. If depression tends to get more or less episodic over time, the burden of recurrent depression will decrease or increase as people get older. More knowledge about the stability of anxiety disorders and MDD from the twenties to the thirties/forties may increase our understanding of the potential impact of such disorders on young adult development. This is important for planning mental health services for young adults.

A limitation in several previous studies of AUD is different diagnostic criteria across waves and use of alcohol abuse as a screen for alcohol dependence [39]. This may affect estimates of prevalence and stability of this disorder [24, 39].

To the best of our knowledge, no previous studies have examined prevalence and stablity in common mental disorders by both: Following the same individuals from the twenties to the thirties/forties, estimating prevalence of anxiety disorders at the different ages including as well as excluding specific phobias, comparing the episodic versus chronic nature of disorders at both ages, and examining comorbidity between disorders at both ages.

### Aims of the study

The overall aim of the current study was to address limitations in previous literature on prevalence and stability of common mental disorders from the twenties to the thirties/forties. More specifically, the aims were to: 1) Estimate the 12-month prevalence of anxiety disorders (with and without specific phobias), mood disorders, and AUD among people aged 19 to 29, 2) Compare these estimates to prevalence estimates ten years later, 3) Compare 12-month to two-week prevalence estimates to examine the episodic nature of disorders across these two developmental periods, 4) Examine the degree to which those who have an anxiety disorder or MDD in the twenties, are at increased risk of having these disorders ten years later, 5) Examine comorbidity of common mental disorders among people in the twenties and ten years later.

## Methods

### Sample

**Fig. 1:**
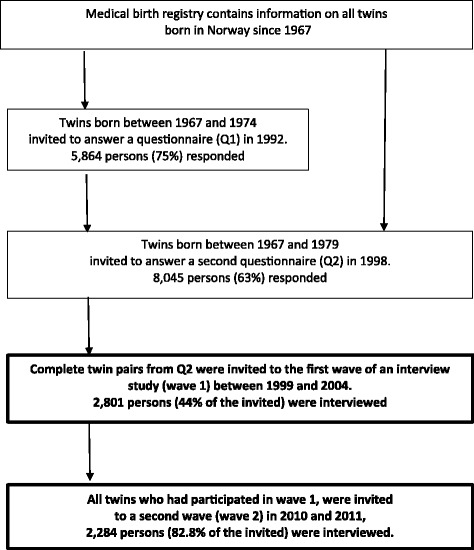
Flow-chart of recruitment to the study. The current study used data from the two interview studies, indicated with bold font

Data came from the Norwegian Institute of Public Health Twin Panel (NIPHTP), which includes health questionnaires at two time points (Q1 and Q2), and diagnostic interviews at two later time points (wave 1 and wave 2). The current study used data from the two interviews. The NIPHTP is described thoroughly elsewhere [40–44], and recruitment is illustrated in Fig. 1.

The first wave of the interview study (wave 1) took place between 1999 and 2004, and 2801 individuals (44% of the invited) were interviewed. All twins who had participated in wave 1 were invited for a re-interview between 2010 and 2011 (wave 2). The follow-up participation rate from wave 1 to wave 2 was 82.8% (*n* = 2284) [41]. Mean age was 28.3 years (range 19–36 years) in wave 1 and 37.9 years (range 30–44 years) in wave 2. Mean time between interviews was 9.6 years (range 7–13 years). Participation in wave 1 was predicted by increasing age and monozygosity, but not by any indicator of mental health [42]. Participation in wave 2 was predicted by high education, female sex, and monozygosity. Neither the total number of mental disorders nor any specific disorder in wave 1 predicted participation in wave 2 [41].

The current study aimed to examine mental disorders in a specific developmental period (19–29 years of age) and follow these individuals over time. Hence, only participants below age 30 in wave 1 were included in the main analyses. This resulted in a sample of 597 men and 1026 women in wave 1, and 457 men and 850 women in wave 2. Mean age in wave 1 was 25.4 years (range 19 to 29), and mean age in wave 2 was 35.3 years (range 30 to 42). Mean time between the two interviews was 9.6 years. Prevalence estimates for the wider age range of the entire sample were also calculated and are presented in Additional file 1: Tables S1 and S2, for readers who are interested in prevalence of mental disorders in a wider age group.

### Measures

A Norwegian version of the computerized Munich Composite International Diagnostic Interview (M-CIDI), originally developed by the World Health Organization, was employed in both interview waves [45]. Diagnoses were coded according to the Diagnostic and Statistical Manual of Mental Disorders, Fourth Edition (DSM-IV). The interview was mainly administered face-to-face in wave 1. For practical reasons, 231 interviews (8.3%) were conducted by telephone. To maximize response rate, all the interviews were conducted by telephone in wave 2. Interviewers were mostly psychology graduate students or experienced psychiatric nurses who had received standardized training by certified instructors. Each twin in a pair was interviewed by a different interviewer.

Respondents answered one question about when they last experienced symptoms of each of the disorders. The response categories were “Within the last two weeks”, “Two weeks to less than a month”, “One to five months”, “6 to 12 months”, “Last 12 months, but cannot remember exactly when”, and “More than one year”. Endorsement of the first option was used to estimate 2-week prevalence, endorsement of any of the first two options to estimate 4-week prevalence, endorsement of any of the first three options to estimate 5(6) month-prevalence, and endorsement of any of the first five options to estimate 12-month-prevalence. Hence, individuals contributing to two-week prevalence estimates were a sub-group of those contributing to all other prevalence estimates of each disorder.

Table 1 shows an overview of combined categories of diagnoses. *AUD* consisted of alcohol abuse and alcohol dependence. *Any mood disorder* consisted of MDD and dysthymia. *Any anxiety disorder W1* consisted of panic disorder, agoraphobia without a history of panic disorder, specific phobias, social phobia, GAD, obsessive-compulsive disorder (OCD), and post-traumatic stress disorder (PTSD). In wave 2, OCD and PTSD were not assessed. These two disorders are no longer classified as anxiety disorders in DSM-5 [46]. *Any anxiety disorder W1–2* did not include these two disorders, and was constructed for both waves to allow comparison of anxiety disorders across age. *Any anxiety disorder W1–2 excluding specific phobias* consisted of all anxiety disorders measured in both waves except specific phobias. *Any mental disorder W1* consisted of *Any mood disorder*, *Any anxiety disorder W1*, and *AUD*. *Any mental disorder W1–2* consisted of *Any mood disorder, Any anxiety disorder W1–2,* and *AUD*. *Any mental disorder W1–2 excluding specific phobias* consisted of *Any mood disorder, Any anxiety disorder W1–2 excluding specific phobias*, and *AUD*.

**Table 1:** Overview of disorders included in the combined categories

Combined category	Includes	Constructed for wave
Any mood disorder	MDD, Dysthymia	1 and 2
Any anxiety disorder W1	Panic disorder, Agoraphobia without a history of panic disorder, Specific phobias, Social phobia, GAD, OCD, PTSD.	1
Any Anxiety disorder W1–2	Panic disorder, Agoraphobia without a history of panic disorder, Specific phobias, Social phobia, GAD.	1 and 2
Any anxiety disorder W1–2 excluding specific phobias	Panic disorder, Agoraphobia without a history of panic disorder, Social phobia, GAD.	1 and 2
AUD	Alcohol abuse, Alcohol dependence.	1 and 2
Any mental disorder W1	Any mood disorder, Any anxiety disorder W1, AUD.	1
Any mental disorder W1–2	Any mood disorder, Any anxiety disorder W1–2, AUD.	1 and 2
Any mental disorder W1–2 excluding specific phobias	Any mood disorder, Any anxiety disorder W1–2 excluding specific phobias, AUD.	1 and 2
*Categories only presented in Supplementary table*		
Drug use disorder	Drug abuse, drug dependence.	1
Any substance use disorder	Drug use disorder, AUD.	1
Any disorder W1- supplement	Any mood disorder, Any anxiety disorder W1, Any substance use disorder	1

*Legends*: *MDD* Major depressive disorder, *GAD* Generalized anxiety disorder, *OCD* Obsessive-compulsive disorder, *PTSD* Post-traumatic stress disorder, *AUD* Alcohol use disorder

*W1* Wave 1, *W2* wave 2

Total as well as sex-stratified prevalence estimates are presented for MDD, *Any mood disorder*, *Any anxiety disorder (W1, W1–2,* as well as *W1–2 excluding specific phobias*), *AUD*, and *Any mental disorder (W1, W1–2*, and *W1–2 excluding specific phobias*). Prevalence estimates for low frequent specific disorders (e.g. panic disorders and dysthymia) are only presented for both genders together. Drug use disorders (drug addiction and drug abuse) were only assess in wave 1, and are presented in Additional file 1: Table S1.

We report 12-month and 2-week prevalence estimates. The other two measured prevalences (5(6)-month and four-week) are presented in Additional file 1: Tables S1 and S2. Interested readers may use those estimates to compare findings with other studies that have used estimates between 12-month and two-week (as for example [22, 47]).

### Statistical analyses

Analyses were performed in Stata version 15. Testing all the prevalence estimates for statistically significant gender differences would imply a large number of tests, thus increasing the risk of random findings. Such testing was therefore only performed for the following estimates: 12-month *Any mental disorder W1–2*, 12-month *Any mood disorder*, 12-month *Any anxiety disorder W1–2*, and 12-month *AUD*.

The 12-month and 2-week prevalence estimates were compared for MDD and *Any anxiety disorder W1–2*. Episodic disorders are expected to show higher 12-month than 2-week prevalences whereas the prevalences are expected to be more similar for more chronic disorders.

Absolute stability was examined by comparing prevalence estimates at the two waves. Only those who participated in wave 2, were included in significance testing of change in prevalence estimates, thus reducing the risk of bias du to potential selective attrition. Relative stability refers to the degree to which it is the same or different people who have a diagnosis across ages [48]. This was examined with logistic regression for MDD, *Any anxiety disorder W1–2*, and *AUD*, adjusted for age in wave 1. We also examined the degree to which MDD in wave 1 predicted having *Any anxiety disorder W1–2* in wave 2, and vice versa, controlled for age. Because of low number of cases and thus low statistical power, *AUD* was not included. Men and women were collapsed in these analyses to increase statistical power.

Comorbidity within each wave was examined with tetrachoric correlations between MDD, *Any anxiety disorders W1–2*, and *AUD*. Comorbidity was also examined by showing the proportion of people with one disorder, who also had another disorder, and by illustrating how many of a hypothetical sample of 100 individuals who would have either one disorder, or a combination of two or three disorders. Men and women were collapsed.

The concordance between twins may affect results. First, standard errors may be too small due to clustering effects [49–51]. This was adjusted for by drawing clusters (twin-pairs) rather than individuals in the bootstrapping/jackknife process and by using the robust estimator for standard errors in logistic regression [49–51]. Second, sensitivity analyses were performed including only one twin from each pair, to see if this affected results.

## Results

### Prevalence estimates

We report results for 597 men and 1026 women (63.2%) who participated in wave 1 (Table 2) and 457 men and 850 women (65.0%) who also participated in wave 2 (Table 3).

**Table 2:** Prevalence of mental disorders in wave 1 (age 19–29 years, *n* = 597 men and 1026 women)

	Men and women		Men		Women		Ratio (women/men) of 12-month prevalence estimates (95% CI)
	12 month prevalence (95% CI)	Two-week prevalence (95% CI)	12 month prevalence (95% CI)	Two-week prevalence (95% CI)	12 month prevalence (95% CI)	Two-week prevalence (95% CI)	
MDD	6.2 5.0–7.4	1.3 0.7–1.8	4.4 2.8–6.3	0.8 0.2–1.6	7.2 5.5–8.8	1.6 0.9–2.4	
Dysthymia	1.7 1.1–2.4	1.4 0.8–2.0					
Any mood disorder ^a^	7.3 5.9–8.6	2.3 1.5–3.0	4.9 3.2–6.8	1.2 0.4–2.2	8.7 6.7–10.6	2.9 1.9–4.0	1.79*** 1.19–2.68
Any anxiety disorder W1 ^b^	20.4 18.3–22.7	15.2 13.5–17.3	9.6 7.0–12.4	5.9 4.2–8.2	26.7 23.7–29.7	20.7 18.1–23.5	
Any anxiety disorder W1–2 ^c^	19.8 17.7–22.0	14.8 13.0–16.7	9.3 7.0–12.3	5.7 4.0–7.9	26.0 23.1–29.0	20.1 17.4–22.7	2.80*** 2.13–3.68
Any anxiety disorder W1–2 excluding specific phobias ^d^	5.3 4.2–6.6	3.2 2.3–4.1	2.5 1.3–4.2	1.3 0.5–2.6	6.9 5.3–8.7	4.2 3.0–5.7	
Specific phobias	16.9 14.9–19.0	12.4 10.8–14.1	7.6 5.5–10.2	4.9 3.3–6.9	22.3 19.5–25.0	16.8 14.5–19.2	
Panic disorder	1.5 1.0–2.2	0.4 0.1–0.7					
Agoraphobia without panic	1.4 0.9–2.0	1.1 0.6–1.7					
Social phobia	2.3 1.7–3.3	1.8 1.2–2.7					
GAD	1.2 0.7–1.9	0.4 0.1–0.7					
OCD	0.3 0.1–0.6	0.2 0.1–0.4					
PTSD	1.2 0.7–1.8	0.7 0.4–1.2					
Alcohol abuse	2.8 2.0–3.7	0.9 0.5–1.5					
Alcohol Dependence	4.7 3.6–5.8	2.1 1.4–2.8					
AUD	6.0 4.8–7.3	2.7 1.9–3.6	8.7 6.5–11.3	3.7 2.3–5.4	4.4 3.2–5.7	2.0 1.2–3.0	0.50*** 0.34–0.74
Any mental disorder W1 ^e^	27.8 25.4–30.3	18.6 16.7–20.6	19.8 16.4–23.4	9.7 7.3–12.4	32.4 29.4–35.7	23.8 21.1–26.6	
Any mental disorder W1–2 ^f^	27.6 25.3–30.2	18.2 16.2–20.1	19.8 16.4–23.4	9.5 7.1–12.2	32.2 29.2–35.4	23.3 20.7–26.0	1.63*** 1.35–1.96
Any mental disorder W1–2 excluding specific phobias ^g^	15.3 13.5–17.5	7.3 6.0–8.8	14.3 11.2–17.3	5.6 3.7–7.7	15.9 13.6–18.5	8.3 6.5–10.3	

*Legends*: *** *p* < 0.001, *MDD* Major depressive disorder, *GAD* Generalized anxiety disorder, *OCD* Obsessive-compulsive disorder, *PTSD* Post-traumatic stress disorder, *AUD* Alcohol use disorders, i.e. alcohol dependence or alcohol abuse

*CI* Confidence interval. 95% CIs are based on standard errors obtained from bootstrapping (1000 replications), bias corrected for skewness in the bootstrap distribution, and adjusted for cluster-effects among twins

^a^MDD and/or dysthymia

^b^Panic disorder, agoraphobia without panic, specific phobias, social phobia, GAD, OCD, and/or PTSD

^c^Panic disorder, agoraphobia without panic, specific phobias, social phobia and/or GAD

^d^Panic disorder, agoraphobia without panic, social phobia and/or GAD

^e^Any mood disorder, Any anxiety disorder W1, AUD

^f^Any mood disorder, Any anxiety disorder W1–2, AUD

^g^Any mood disorder, Any anxiety disorder W1–2 excluding specific phobias, AUD

**Table 3:** Prevalence of mental disorders in wave 2 (age 30–42 years, *n* = 457 men and 850 women)

	Men and women		Men		Women		Ratio (women/men) of 12-month prevalence estimates (95% CI)
	12 month prevalence (95% CI)	Two-week prevalence (95% CI)	12 month prevalence (95% CI)	Two-week prevalence (95% CI)	12 month prevalence (95% CI)	Two-week prevalence (95% CI)	
MDD	5.0 3.8–6.3	1.1 0.6–1.7	3.3 1.9–5.2	0.9 0.2–1.9	5.9 4.3–7.5	1.2 0.6–2.0	
Dysthymia	1.9 1.2–3.0	0.9 0.5–1.5					
Any mood disorder^a^	5.8 4.6–7.3	1.6 1.0–2.4	3.7 2.3–5.9	1.1 0.2–2.3	7.0 5.3–8.7	1.9 1.1–3.0	1.87** 1.10–3.17
Any anxiety disorder W1–2^b^	16.1 13.9–18.4	8.3 6.8–9.7	5.9 4.0–8.2	2.9 1.5–4.7	21.6 18.6–24.6	11.2 9.0–13.2	3.63*** 2.46–5.35
Any anxiety disorder W1–2 excluding specific phobias^c^	6.5 5.2–8.0	2.6 1.8–3.7	2.6 1.3–4.3	0.9 0.2–1.9	8.5 6.7–10.7	3.5 2.4–5.0	
Specific phobias	12.3 10.3–14.3	6.5 5.2–7.9	4.1 2.4–6.1	2.2 1.0–3.9	16.8 14.1–19.5	8.8 6.9–10.8	
Panic disorder	2.1 1.4–3.0	0.5 0.2–1.1					
Agoraphobia without panic	1.2 0.6–1.9	0.6 0.2–1.2					
Social phobia	3.9 2.9–5.2	1.4 0.7–2.1					
GAD	1.5 0.9–2.2	0.5 0.2–0.9					
AUD	2.7 1.8–3.7	1.2 0.7–1.8	5.3 3.2–7.2	2.4 1.2–4.0	1.3 0.7–2.2	0.5 0.1–1.0	0.25** 0.12–0.50
Any mental disorder W1–2^d^	20.9 18.6–23.3	10.0 8.3–11.7	12.9 9.8–16.3	5.8 3.8–8.1	25.3 22.1–28.6	12.3 10.3–14.5	1.96*** 1.50–2.55
Any mental disorder W1–2 excluding specific phobias^e^	12.4 10.5–14.4	4.9 3.6–6.2	10.0 7.3–12.6	4.2 2.5–6.2	13.7 11.4–16.3	5.2 3.7–6.9	

*Legends*: ** *p* < 0.01, *** *p* < 0.001, *MDD* Major depressive disorder, *GAD* Generalized anxiety disorder, *AUD* Alcohol use disorders, i.e. alcohol dependence or alcohol abuse

*CI* Confidence interval. 95% CIs are based on standard errors obtained from bootstrapping (1000 replications), bias corrected for skewness in the bootstrap distribution and adjusted for cluster-effects among twins

^a^MDD and/or dysthymia

^b^Panic disorder, agoraphobia without panic, specific phobias, social phobia and/or GAD

^c^Panic disorder, agoraphobia without panic, social phobia and/or GAD

^d^Any mood disorder, Any anxiety disorder W1–2, AUD

^e^Any mood disorder, Any anxiety disorder W1–2 excluding specific phobias, AUD

In wave 1, when the participants were in the twenties, *Any anxiety disorder W1* was the most prevalent combined category of disorders for both men and women (see Table 2). The 12-month prevalence for *Any anxiety disorder W1* was 9.6% (95% confidence interval (CI) = 7.0–12.4) for men and 26.7% (95% CI = 23.7–29.7) for women. For *Any anxiety disorder W1–2*, the 12-month prevalence was 9.3 (95% CI = 7.0–12.3) for men and 26.0% (95% CI = 23.1–29.0) for women. *AUD* ranked second among men (8.7% 12-month prevalence, 95% CI = 6.5–11.3), and *Any mood disorder* ranked second among women (8.7% 12-month prevalence, 95% CI = 6.7–10.6). Specific phobias were highly prevalent among men and women (12-month prevalence = 7.6% with 95% CI = 5.5–10.2 for men and 22.3% with 95% CI = 19.5–25.0 for women). *AUD* and *Any mood disorder* were the most common groups of disorders for men, and *Any mood disorder* was the most common group of disorders for women when specific phobias were excluded. Results from significance testing of gender difference showed that *Any mental disorder W1–2*, *Any anxiety disorder W1–2*, and *Any mood disorder* were more prevalent among women than men, while *AUD* was more prevalent among men than women (see Table 2).

In wave 2, the 12-month prevalence of *Any anxiety disorder W1–2* was 5.9% (95% CI = 4.0–8.2) for men and 21.6% (95% CI = 18.6–24.6) for women (see Table 3). Again, this was mainly due to the high prevalence of specific phobias (12-month prevalence was 4.1% with 95% CI = 2.4–6.1 for men and 16.8% with 95% CI = 14.1–19.5 for women). *Any mood disorder* was the second most prevalent group of disorders among women (12-month prevalence 7.0% with 95% CI = 5.3–8.7), and *AUD* was the second most common disorder among men (12-month prevalence = 5.3% with 95% CI = 3.2–7.2). *Any mental disorder W1–2*, *Any mood disorder,* and *Any anxiety disorder W1–2,* were more prevalent among women than men, and the reverse was true for *AUD* (see Table 3).

### Stability

There was a reduction in *Any mental disorder W1–2*, Specific phobias, and *AUD* from wave 1 to wave 2 (95% CI for differences did not include zero). Estimates were stable for MDD and *Any anxiety disorder W1–2 excluding specific phobias* (95% CI for difference included zero)*.*

Relative stability was measured with logistic regression, presented in Table 4. Having MDD, *Any anxiety disorder W1–2,* or *AUD* in the twenties, increased the risk of having these disorders ten years later. The results showed that *Any anxiety disorder W1–2* in wave 1 predicted MDD in wave 2, even controlled for MDD in wave 1.

**Table 4:** Relative stability in mental disorders from age 19–29 to age 30–42 estimated with logistic regression

	Disorder in W1 not adjusted for each other OR (95% CI)	MDD and Anxiety in W1 mutually adjusted for each other OR (95% CI)
*MDD in wave 2*		
MDD in wave 1	3.56*** (1.81–6.98)	2.60** (1.31–5.17)
Anxiety in wave 1	2.61*** (1.54–4.42)	2.18** (1.26–3.75)
*Anxiety in wave 2*		
MDD in wave 1	3.94*** (2.36–6.60)	1.77 (0.96–3.26)
Anxiety in wave 1	9.01*** (6.45–12.59)	8.33*** (5.90–11.78)
*AUD in wave 2*	8.10***	
AUD in wave 1	(3.69–17.76)	

*Legends*: *OR* Odds ratio, *CI* Confidence interval, *MDD* Major depressive disorder, Anxiety = *Any anxiety disorder W1–2 (*Panic disorder, agoraphobia without panic, specific phobias, social phobia and/or generalized anxiety disorder (GAD))*.* Standard errors were obtained from the robust estimator in Stata, accounting for cluster-effects among twins

Men and women were collapsed in the analyses to increase statistical power. All analyses were adjusted for age in wave 1

Despite relative stability from wave 1 to wave 2, 82.4% of those who had MDD, 54.9% of those who had *Any anxiety disorder W1–2*, and 85.4% of those who had *AUD* in Wave 1 (12-mont prevalence), did not have the same disorder in wave 2.

### 12-month versus 2-week prevalence estimates

The 12-month prevalence of MDD was significantly higher than the 2-week prevalence in both waves. The 12-month to two-week ratios for MDD was 4.8 (95% CI = 3.9–7.5) in wave 1 and 4.6 (95% CI = 3.1–8.6) in wave 2. For *Any anxiety disorder W1–2* the ratios were 1.3 (95% CI = 1.3–1.4) in wave 1 and 1.9 (95% CI = 1.7–2.2) in wave 2, indicating a more episodic nature of MDD than anxiety disorders.

### Comorbidity

Tetrachoric correlations between 12-month MDD, *Any anxiety disorder W1–2*, and *AUD* within each wave are presented in Table 5. There was a strong association between MDD and *Any anxiety disorder W1–2* in both waves.

**Table 5:** Tetrachoric correlations between different disorders at wave 1 (age 19–29) and wave 2 (age 30–42)

		Wave 1			Wave 2		
		MDD	Anxiety W1–2^a^	AUD	MDD	Anxiety W1–2^a^	AUD
Wave 1	MDD		0.45***	0.00	0.31**	0.38***	0.16
	Anxiety W1–2^a^			0.10	0.26**	0.65***	0.02
	AUD				0.07	0.07	0.49***
Wave 2	MDD					0.54***	0.12
	Anxiety W1–2^a^						0.03
	AUD						

*Legends*: ^a^Panic disorder, agoraphobia without panic, specific phobias, social phobia and/or generalized anxiety disorder (GAD)

*MDD* Major depressive disorder. *AUD* Alcohol use disorders, i.e. alcohol dependence or alcohol abuse. Standard errors were obtained from the jackknife estimator, accounting for cluster-effects among twins. **p < 0.01, ***p < 0.001**.** Men and women were collapsed to increase statistical power

**Fig. 2:**
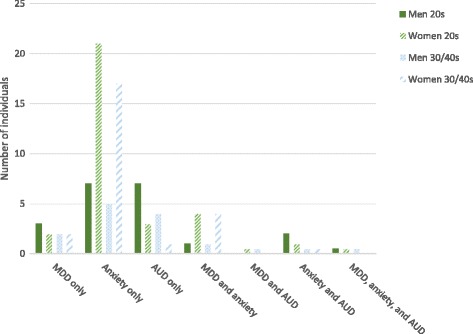
Combinations of disorders in hypothetical groups of 100 men and 100 women at different ages. Legend: MDD = major depressive disorder, Anxiety = Any anxiety disorder W1–2 (Panic disorder, agoraphobia without panic, specific phobias, social phobia and/or generalized anxiety disorder (GAD)), AUD = alcohol use disorder (i.e. alcohol dependence and alcohol abuse)

Comorbidity was also examined by calculating how many of those who had one disorder, also had another disorder. The results are presented in Table 6. The comorbidity numbers for *AUD* should be interpreted with caution in wave 2, as few people had this diagnosis. Figure 2 shows the number of people with pure versus comorbid MDD, *Any anxiety disorder W1–2,* and *AUD* in hypothetical samples of 100 men and 100 women in the twenties and in the thirties/forties.

**Table 6:** Comorbidity wave 1 (age 19–29) and wave 2 (age 30–42)

	Men			Women		
	% with comorbid MDD	% with comorbid Anxiety W1–2 ^a^	% with comorbid AUD	% with comorbid MDD	% with comorbid Anxiety W1–2^a^	% with comorbid AUD
*Wave 1*						
MDD		34.6 17.6–55.6	3.8 0–14.3		66.2 54.8–76.8	6.8 1.5–13.7
Anxiety W1–2^a^	15.8 7.3–26.8		17.5 8.5–27.3	17.4 13.1–21.9		5.9 3.6–9.0
AUD	1.9 0–7.1	17.5 8.5–27.3		11.1 3.4–21.1	35.6 22.2–50.0	
*Wave 2*						
MDD		33.3 13.0–53.6	16.7 3.7–31.8		57.0 46.5–67.0	2.0 0.0–5.4
Anxiety W1–2^a^	17.4 8.3–31.1		8.7 2.0–18.0	19.5 14.8–24.3		1.7 0.3–3.4
AUD	11.4 2.9–23.3	11.8 3.0–23.5		14.3 0.0–37.5	7.1 0.0–25.0	

*Legends*: *MDD* Major depressive disorder, *AUD* Alcohol use disorders, i.e. alcohol dependence or alcohol abuse

^a^Panic disorder, agoraphobia without panic, specific phobias, social phobia and/or generalized anxiety disorder (GAD)

### Sensitivity analyses of concordance between twins

As mentioned in the methods section, the use of twins may have affected results, and sensitivity analyses were therefore performed. If one twin did not have any mental disorder during the last 12 months in wave 1, the co-twin’s risk of having a mental disorder was 21.7% (compared to 27.8% in the full sample). When one twin had a mental disorder, the co-twin’s risk was 43.7%. This concordance between twins was expressed in an intraclass correlation of 0.21. Prevalence of MDD, *Any anxiety disorders W1*, *AUD*, and *Any mental disorder W1* were estimated for a sub-sample where one twin was randomly chosen from each pair (see Table 7). The estimates were quite similar to estimates from both twins. The ratio of the results (estimates from the entire sample/estimates from only one twin) varied between 1.00 and 1.04.

**Table 7:** Prevalence estimates in wave 1 when using both twins versus one twin in each pair

	12-month prevalence estimate using both twins 95% CI^c^	12-month prevalence estimate only including one twin from each pair 95% C.I.	Ratio Entire sample estimate/Estimate using one twin from each pair
MDD	6.2 5.0–7.4	6.2 4.5–8.0	1.00
Any anxiety disorder W1^a^	20.4 18.3–22.7	19.6 17.2–22.5	1.04
AUD	6.0 4.8–7.3	5.8 4.4–7.5	1.03
Any mental disorder W1^b^	27.8 25.4–30.3	27.8 24.7–31.0	1.00

*Legends*: *CI* Confidence interval, *MDD* Major depressive disorder

^a^Panic disorder, agoraphobia without panic, specific phobias, social phobia, generalized anxiety disorder (GAD), obsessive-compulsive disorder (OCD), and/or post-traumatic stress disorder (PTSD)

*AUD* Alcohol use disorders, i.e. alcohol dependence or alcohol abuse

^b^Any mental disorder W1 = Any mood disorder, Any anxiety disorder W1, AUD

^c^95% CIs are based on standard errors obtained from bootstrapping (1000 replications), bias corrected for skewness in the bootstrap distribution, and adjusted for cluster-effects among twins

## Discussion

MDD, anxiety disorders, and AUD were highly prevalent at age 19–29. The prevalence of mental disorders was lower at age 30–42, and this was mainly due to decrease in AUD and specific phobias. Prevalence estimates of MDD and anxiety disorders excluding specific phobias were stable in this period. Individuals who had MDD, an anxiety disorder, or AUD in the twenties were of increased risk of having these disorders in the thirties/forties compared to those who did not have these diagnoses in the twenties.

### Prevalence estimates

The 12-month prevalence of depressive disorders in people in the twenties was lower in the current study compared to young adults in the CICS [16], the Dunedin study, NZMHS, NCS, and NCS-R [21, 23], but similar to the prevalence among 26-year-olds in the Simmons longitudinal study [24]. The current findings were very similar to results from these other studies with regard to prevalence of anxiety disorders in the twenties. In the Dunedin study, alcohol dependence among people aged 18 to 32 years was more prevalent than the broader category of AUD in both waves in the current study [21]. Likewise, the narrower category alcohol abuse was more prevalent in TRAILS and NCS-R than AUD in the current sample [22, 23]. In the Simmons longitudinal study, the prevalence of AUD was similar to the current results [24].

Our findings are in line with previous studies showing that anxiety disorders tend to be the most prevalent group of disorders, both in the general adult population and among young adults [21, 52]. The current results are in line with previous Norwegian findings of higher prevalence of specific phobias compared to international studies [6, 53].

The most prevalent groups of disorders were mood disorders and AUD among men and mood disorders among women when specific phobias were excluded. Such phobias are very common, but by definiton highly situation-specific. The current results allowed differentiating between this very circumscrbied disorder and mental disorders that affect people in more complex ways across different situations (e.g. MDD, GAD, and AUD).

### Stability

Mental disorders became less prevalent as participants went from the twenties to the thirties/forties. Our results converge with findings from the Simmons Longitudinal study regarding a decrease in specific phobias and AUD [24]. Other anxiety disorders did not become less prevalent in the current study. Findings from the Dunedin study showed a marked decrease in acohol dependence, and quite stable prevalence of MDD and anxiety disorders (including specific phobias, GAD, panic disorder, agoraphobia, OCD, and PTSD) from age 21 to age 32 [21]. Decrease in AUD across young adulthood may reflect the circumstances of people in the twenties, who often do not have responsibility for children, and many are students.

Results from a previous study indicate that a third of those who met diagnostic criteria for AUD in late adolescence, also met criteria for AUD at age 26 [54]. In the current study, only about 15% of those who met diagnostic criteria for AUD in the twenties also had AUD in the thirties/forties. Hence, the course of AUD seems to be less chronic from the twenties to the thirties/forties than from late adolescence to age 26. Nevertheless, the current results showed that those who had AUD in the twenties, had increased risk of having AUD in the thirties/forties compared to those who did not have AUD in the twenties.

Previous studies have found that the majority of individuals with anxiety and depressive disorders at one time point are free from these disorders after three to 11 years [25–27, 55]. This was also the case in the current study. However, those who had MDD or anxiety disorders in the twenties, had increased risk of having these disorders in the thirties/forties compared to those who did not have the disorders in the twenties. This is in line with previous findings from a wider age group of adults (age 18 to 65 at baseline) [56].

Anxiety disorders in the twenties strongly predicted MDD ten years later, even controlling for MDD in the twenties, in line with findings of stable and unspecific genetic risk of mental disorders [34, 35, 57]. The association between MDD in the twenties and anxiety disorders in the thirties/forties, controlled for anxiety disorders in the twenties, was not statistically significant, which may be due to low statistical power. However, others have reported similar results by showing that baseline GAD predicted persistence of MDD, but not the other way around among adolescents and young adults in the NCS-2 [31]. This is also in line with other longitudinal findings from the general adult population, showing that anxiety at one time point predicted depression at a later time to a stronger degree than the reverse [58]. In the Netherlands Mental Health Survey and Incidence Study (NEMESIS), mood disorder at follow-up was predicted by anxiety disorders three years earlier, and incident anxiety disorder was predicted by previous mood disorders [59]. In the US National Epidemiological Survey on Alcohol and Related Conditions (NESARC), there was reciprocal longitudinal associations between incidence of MDD and GAD [60]. The Finnish Health 2011 study reported that baseline anxiety disorder predicted new-onset MDD at 11-year follow-up [61]. Hence, anxiety disorders and MDD seem to have common risk factors, but it is unclear whether anxiety is a more robust predictor of later MDD than the reverse.

MDD seemed to be more episodic in its nature than anxiety disorders, in line with previous studies [22, 55]. There was no change toward a more or less chronic course of any of these disorders when the participants got older.

### Comorbidity

There was substantial comorbidity between disorders within each wave, in line with previous research [4, 7, 32, 37], and no evidence of increased or decreased comorbidity between MDD and anxiety disorders from the twenties to the thirties/forties. In both waves, more than half of women and about one third of men with MDD, also had an anxiety disorder. Among those who had an anxiety disorder, one in five or fewer, also had MDD. Young people with vulnerability of both MDD and anxiety, may at any time point have higher risk of experiencing a current anxiety disorder than current MDD because the latter disorder tends to be more episodic.

### Implications for policy makers

Previous research has shown that most people with mental disorders go untreated [62, 63]. The current results highlight the need to make adequate treatment easily available for young adults, to help them cope with their developmental tasks (e.g. attain education and work experience). Easily available treatment is important because of the high prevalence of mental disorders in the twenties and the thirties/forties, and because these disorders were long lasting and/or recurrent across ten years for a substantial number of young adults.

The high prevalence of AUD between age 19 and 29 suggests that it is important to implement alcohol interventions particularly tailored to fit this age group and their often-changing life circumstances. Specific phobias may impose functional impairment only in particular situations, but may be serious if those situations are difficult to avoid. There seems to be a particularly high need for efficient treatment of specific phobias for people in the twenties.

Other anxiety disorders and MDD did not become less prevalent with age. Anxiety disorders and MDD did not become more episodic from the twenties to the thirties/forties, and comorbidity between them did not change during this period. Hence, mental health needs for other disorders than AUD and specific phobias are stable from the twenties to the thirties/forties, and many young adults will need treatment for more than one mental disorder.

As noted by Schaefer and colleagues, public knowledge about the very common nature of mental disorders may contribute to reduced stigma [64]. In line with this, public information about the current findings of high prevalence of mental disorders among young adults may contribute to reduced stigma in this age group.

### Strengths and limitations

The major strength of the present study is the large population-based sample, which has been assessed twice across 10 years for DSM-IV disorders by structured interviews. However, some limitations warrant consideration. First, the results may not be generalizable to other age groups. Second, there may be bias in the results due to selection in recruitment and attrition at follow-up. People with mental health problems tend to be under-represented in survey studies [65]. However, as discussed above, mental health variables did not predict participation in either of the interview waves [41, 42], and bias due to selective response may thus not be substantial.

Third, the results are based on a sample of twins, which may reduce generalizability to the general population. A study from the US compared self-reported symptoms of depression, panic-phobia, somatization, and insomnia between twins and their non-twin relatives, and found that twins had significantly, but modestly, higher scores on the panic-phobia factor, but reported similar levels of psychiatric symptoms as their non-twin relatives on the other factors [66]. The authors concluded that *“twins are typical for the general non-twin population in their risk for psychiatric symptoms and syndromes.”* (p. 590). Nevertheless, the current findings may be affected by the fact that two individuals from each family were recruited to the study. There was substantial concordance between two twins in a pair regarding mental disorders. Sensitivity analyses were performed with only one twin from each pair included, and results were very similar to the results from both twins.

## Conclusion

Mental disorders are common among young people in the twenties and the thirties/forties, but AUD and specific phobias seem to become less prevalent over this age period. Nevertheless, some people are at enduring increased risk of impaired functioning due to mental disorders in consecutive developmental periods. Policy makers should ensure that mental health interventions are available and tailored for young people to help them master their developmental tasks.

## Additional file

Additional file 1: **Tables S1 and S2.** Prevalence estimates not shown in the main files. (DOCX 30 kb)

## Abbreviations

AUD: Alcohol use disorders
CI: Confidence interval
CICS: Children in Community Study
DSM-IV: Diagnostic and Statistical Manual of Mental Disorders, Fourth Edition
GAD: Generalized anxiety disorder
GBD: Global Burden of Disease
M-CIDI: Munich Composite International Diagnostic Interview
MDD: Major depressive disorder
NCS: US National Comorbidity Survey
NCS-2: US National Comorbidity Survey follow-up
NCS-R: US National Comorbidity Survey Replication
NEMESIS: Netherlands Mental Helath Survey and Incidence Study
NESARC: National Epidemiological Survey on Alcohol and Related Conditions
NIPHTP: Norwegian Institute of Public Health Twin Panel
NZMHS: New Zealand Mental Health Survey
OCD: Obsessive-compulsive disorder
PTSD: Post-traumatic stress disorder
Q1: Questionnaire 1 in 1992
Q2: Questionnaire 2 in 1998
TRAILS: Tracking Adolescents’ Individual Lives Survey
YLD: Years lived with disability
