# Supplementary material for: Prevalence and stability of mental disorders among young adults: findings from a longitudinal study
Source: BMC Psychiatry. 2018 Mar 12;18:65. doi: 10.1186/s12888-018-1647-5 (PMC5848432; doi:10.1186/s12888-018-1647-5)
Supplement: Supplementary file 1 — Tables S1 and S2. Prevalence estimates not shown in the main files. (DOCX 30 kb) [file 12888_2018_1647_MOESM1_ESM.docx]

Additional file

Table S1. Wave-1-prevalences in the entire sample (N=1,024 men and 1,777 women, age 19-36 years).

|  | Men | | | |  | | Women | | | | | |
| --- | --- | --- | --- | --- | --- | --- | --- | --- | --- | --- | --- | --- |
|  | 12-month prevalence  95%.CI | 6-month prevalence  95%.CI | 4-week prevalence  95%.CI | Two-week prevalence  95%.CI | |  | 12-month prevalence  95%.CI | 6-month prevalence  95%.CI | 4-week prevalence  95%.CI | | Two-week prevalence  95%.CI | |
|  |  |  |  |  |  | |  |  | |  | |  |
| MDD | 5.0%  3.7-6.4 | 2.8%  1.9-3.8 | 1.3%  0.7-2.2 | 0.9%  0.4-1.5 |  | | 6.8%  5.6-8.0 | 4.5%  3.5-5.5 | | 2.1%  1.4-2.8 | | 1.6%  1.0-2.2 |
| Dysthymia | 1.2%  0.6-2.0 | 1.0%  0.4-1.7 | 0.9%  0.4-1.6 | 0.9%  0.4-1.6 |  | | 1.9%  1.3-2.6 | 1.8%  1.2-2.5 | | 1.6%  1.1-2.3 | | 1.5%  1.0-2.2 |
| Any mood disorder^a)^ | 5.7%  4.4-7.5 | 3.3%  2.4-4.7 | 2.0%  1.2-2.9 | 1.6%  0.9-2.4 |  | | 8.1%  6.7-9.4 | 5.8%  4.6-6.9 | | 3.4%  2.6-4.3 | | 2.8%  2.0-3.6 |
| Panic disorder | 0.9%  0.4-1.7 | 0.5%  0.1-1.0 | 0.3%  0.1-0.7 | 0.1%  0.0-0.4 |  | | 1.9%  1.3-2.6 | 1.7%  1.1-2.4 | | 1.1%  0.6-1.6 | | 0.8%  0.4-1.2 |
| Agoraphobia without panic | 0.9%  0.4-1.6 | 0.7%  0.2-1.4 | 0.7%  0.2-1.4 | 0.6%  0.2-1.3 |  | | 1.9%  1.3-2.7 | 1.7%  1.1-2.3 | | 1.6%  1.1-2.3 | | 1.4%  0.9-2.0 |
| Specific phobias | 7.0%  5.4-8.6 | 5.9%  4.5-7.5 | 5.2%  3.9-6.8 | 4.7%  3.5-6.2 |  | | 20.9%  18.7-22.8 | 19.0%  17.1-20.9 | | 16.6%  14.7-18.5 | | 15.7%  13.8-17.4 |
| Social phobia | 1.3%  0.7-2.1 | 1.0%  0.5-1.7 | 1.0%  0.5-1.7 | 0.8%  0.4-1.5 |  | | 3.1%  2.2-3.9 | 2.9%  2.0-3.8 | | 2.6%  1.8-3.5 | | 2.4%  1.6-3.3 |
| GAD | 0.4%  0.1-0.8 | 0.2%  0.0-0.5 | 0.1%  0.0-0.4 | 0.1%  0.0-0.4 |  | | 1.4%  0.9-2.0 | 0.9%  0.5-1.4 | | 0.6%  0.3-1.0 | | 0.5%  0.2-0.8 |
| OCD | 0.2%  0.0-0.6 | 0.2%  0.0-0.6 | 0.2%  0.0-0.6 | 0.2%  0.0-0.6 |  | | 0.3%  0.1-0.7 | 0.3%  0.1-0.6 | | 0.2%  0.1-0.5 | | 0.2%  0.1-0.5 |
| PTSD | 0.4%  0.1-0.9 | 0.3%  0.0-0-7 | 0.2%  0.0-0.5 | 0.2%  0.0-0.5 |  | | 1.5%  0.9-2.0 | 1.3%  0.8-1.8 | | 1.1%  0.7-1.7 | | 1.1%  0.7-1.6 |
| Any anxiety disorder W1^b)^ | 9.4%  7.6-11.4 | 7.6%  6.0-9.4 | 6.8%  5.4-8.6 | 5.9%  4.6-7.6 |  | | 25.3%  23.0-27.6 | 23.2%  21.0-25.4 | | 20.6%  18.4-22.8 | | 19.5%  17.3-21.5 |
| Any anxiety disorder W1-2^c)^ | 9.1%  7.4-11.1 | 7.4%  5.8-9.1 | 6.6%  5.2-8.4 | 5.7%  4.5-7.4 |  | | 24.6%  22.2-26.7 | 22.6%  20.4-24.7 | | 20.1%  17.8-22.0 | | 18.9%  16.7-20.8 |
| Any substance use disorder^d)^ | 7.1%  5.6-8.9 | 5.8%  4.4-7.3 | 3.7%  2.5-4.9 | 2.8%  1.7-3.8 |  | | 2.9%  2.2-3.7 | 2.5%  1.7-3.2 | | 1.5%  1.0-2.1 | | 1.5%  0.9-2.1 |
| Alcohol abuse | 3.5%  2.4-4.7 | 2.4%  1.5-3.4 | 1.3%  0.7-2.0 | 1.1%  0.6-1.7 |  | | 1.1%  0.7-1.7 | 1.0%  0.5-1.5 | | 0.4%  0.1-0.7 | | 0.4%  0.1-0.7 |
| Alcohol Dependence | 5.2%  3.8-6.7 | 4.3%  3.0-5.8 | 2.9%  1.9-4.1 | 2.1%  1.2-3.1 |  | | 2.3%  1.6-3.0 | 1.9%  1.3-2.6 | | 1.2%  0.8-1.8 | | 1.2%  0.7-1.7 |
| AUD | 6.7%  5.2-8.4 | 5.5%  4.2-7.1 | 3.5%  2.4-4.7 | 2.7%  1.7-3.8 |  | | 2.9%  2.1-3.7 | 2.5%  1.8-3.3 | | 1.5%  1.0-2.1 | | 1.5%  1.0-2.1 |
| Drug abuse | 0.6%  0.2-1.2 | 0.6%  0.2-1.2 | 0.3%  0.1-0.7 | 0.2%  0.1-0.5 |  | | 0.1%  0.0-0.3 | 0.0% | | 0.0% | | 0.0% |
| Drug Dependence | 0.6%  0.2-1.1 | 0.3%  0.1-0.7 | 0.3%  0.1-0.7 | 0.3%  0.1-0.7 |  | | 0.0% | 0.0% | | 0.0% | | 0.0% |
| Any drug disorder | 0.9%  0.3-1.6 | 0.7%  0.2-1.4 | 0.4%  0.1-0.9 | 0.3%  0.1-0.7 |  | | 0.1%  0.0-0.3 | 0.0% | | 0.0% | | 0.0% |
| Central stimulating  dependence | 0.1%  0.1-0.2 | 0.1%  0.1-0.2 | 0.1%  0.1-0.2 | 0.1%  0.1-0.2 |  | |  |  | |  | |  |
| Cannabis dependence | 0.6%  0.2-1.1 | 0.3%  0.1-0.7 | 0.3%  0.1-0.9 | 0.3%  0.1-0.7 |  | |  |  | |  | |  |
| Cocaine dependence | 0.1%  0.1-0.2 | 0.1%  0.1-0.2 | 0.1%  0.1-0.2 | 0.0% |  | |  |  | |  | |  |
| Any mental disorder^e)^ | 18.7%  16.2-21.3 | 14.6%  12.3-16.8 | 11.0%  8.9-13.0 | 9.3%  7.4-11.1 |  | | 30.3%  28.1-32.8 | 27.4%  25.1-29.7 | | 23.2%  21.3-25.5 | | 21.9%  19.9-24.0 |

Legends: MDD = major depressive disorder. AUD = alcohol use disorders, i.e. alcohol dependence or alcohol abuse.

CI = confidence interval. 95% CIs are based on standard errors obtained from bootstrapping (1000 replications), bias corrected and adjusted for cluster-effects among twins.

^a)^ MDD and/or dysthymia

^b)^ Panic disorder, agoraphobia without panic, specific phobias, social phobia, GAD, OCD, and/or PTSD.

^c)^ Panic disorder, agoraphobia without panic, specific phobias, social phobia and/or GAD.

^d)^ Alcohol abuse, alcohol dependence, drug abuse and/or drug dependence.

^e)^ Any mood disorder, Any anxiety disorder W1 and/or any substance use disorder.

Table S2. Wave-2-prevalences in the entire sample (N=802 men and 1,482 women, age 30-44 years).

|  | Men | | | |  | Women | | | |
| --- | --- | --- | --- | --- | --- | --- | --- | --- | --- |
|  | 12-month prevalence  95%.CI | 6-month prevalence  95%.CI | 4-week prevalence  95%.CI | Two-week prevalence  95%.CI |  | 12-month prevalence  95%.CI | 6-month prevalence  95%.CI | 4-week prevalence  95%.CI | Two-week prevalence  95%.CI |
|  |  |  |  |  |  |  |  |  |  |
| MDD | 3.0%  1.9-4.2 | 1.9%  1.0-2.9 | 1.3%  0.5-2.0 | 0.8%  0.2-1.4 |  | 6.8%  5.6-8.3 | 4.7%  3.6-5.8 | 2.3%  1.5-3.2 | 1.6%  0.9-2.2 |
| Dysthymia | 1.1%  0.5-2.0 | 0.9%  0.4-1.6 | 0.5%  0.1-1.1 | 0.4%  0.0-0.9 |  | 2.2%  1.4-3.0 | 2.0%  1.2-2.7 | 1.5%  0.9-2.2 | 1.4%  0.8-2.1 |
| Any mood disorder^a)^ | 3.6%  2.4-4.9 | 2.4%  1.4-3.5 | 1.5%  0.8-2.4 | 0.9%  0.4-1.6 |  | 7.8%  6.4-9.4 | 5.5%  4.2-6.7 | 3.1%  2.2-4.1 | 2.4%  1.6-3.3 |
| Panic disorder | 0.8%  0.2-1.5 | 0.4%  0.0-0.9 | 0.1%  0.0-0.5 | 0.1%  0.0-0.5 |  | 2.4%  1.6-3.2 | 2.0%  1.3-2.7 | 1.2%  0.7-1.9 | 0.7%  0.3-1.1 |
| Agoraphobia without panic | 0.3%  0.0-0.7 | 0.3%  0.0-0.7 | 0.1%  0.0-0.5 | 0.1%  0.0-0.5 |  | 1.6%  1.0-2.3 | 1.2%  0.7-1.8 | 1.0%  0.5-1.6 | 1.0%  0.5-1.6 |
| Specific phobias | 4.0%  2.7-5.4 | 3.3%  2.1-4.6 | 2.4%  1.5-3.5 | 2.3%  1.3-3.3 |  | 15.7%  13.8-17.8 | 12.5%  10.7-14.3 | 9.3%  7.9-10.9 | 8.3%  7.1-9.9 |
| Social phobia | 1.4%  0.6-2.2 | 1.4%  0.6-2.2 | 0.9%  0.4-1.6 | 0.8%  0.3-1.4 |  | 4.4%  3.3-5.6 | 3.5%  2.6-4.6 | 2.0%  1.3-2.9 | 1.8%  1.1-2.5 |
| GAD | 0.4%  0.0-0.9 | 0.4%  0.0-0.9 | 0.1%  0.0-0.5 | 0.0% |  | 2.1%  1.4-2.9 | 1.6%  1.0-2.3 | 0.9%  0.5-1.4 | 0.8%  0.4-1.3 |
| Any anxiety disorder W1-2^b)^ | 5.8%  4.2-7.4 | 4.7%  3.1-6.2 | 3.4%  2.2-4.8 | 3.1%  1.9-4.6 |  | 20.0%  17.9-22.4 | 16.4%  14.5-18.6 | 11.8%  10.1-13.5 | 10.5%  8.9-12.3 |
| Alcohol abuse | 1.8%  0.9-2.9 | 1.0%  0.5-1.8 | 0.4%  0.1-1.0 | 0.4%  0.1-1.0 |  | 0.4%  0.1-0.8 | 0.3%  0.1-0.7 | 0.1%  0.0-0.3 | 0.1%  0.0-0.3 |
| Alcohol Dependence | 3.1%  2.0-4.4 | 2.6%  1.6-3.8 | 1.9%  1.0-2.9 | 1.6%  0.9-2.7 |  | 0.6%  0.3-1.1 | 0.5%  0.2-0.9 | 0.3%  0.1-0.6 | 0.2%  0.0-0.5 |
| AUD | 4.4%  3.0-5.9 | 3.5%  2.3-4.8 | 2.1%  1.2-3.2 | 1.9%  1.1-2.8 |  | 0.9%  0.5-1.5 | 0.7%  0.4-1.3 | 0.4%  0.1-0.8 | 0.3%  0.1-0.7 |
| Any mental disorder^c)^ | 12.0%  9.6-14.4 | 9.1%  7.2-11.3 | 6.1%  4.4-8.0 | 5.2%  3.7-6.9 |  | 24.0%  21.7-26.4 | 19.2%  17.0-21.4 | 13.5%  11.7-15.2 | 11.8%  10.1-13.5 |

Legends: MDD = major depressive disorder. AUD = alcohol use disorders, i.e. alcohol dependence or alcohol abuse.

CI = confidence interval. 95% CIs are based on standard errors obtained from bootstrapping (1000 replications), bias corrected and adjusted for cluster-effects among twins.

^a)^ MDD and/or dysthymia

^b)^ Panic disorder, agoraphobia without panic, specific phobias, social phobia and/or GAD.

^c)^ Any mood disorder, Any anxiety disorder W1-2, and/or AUD.
